# Supplementary material for: Functional Dissection of Leishmania major Membrane Components in Resistance to Cholesterol-Dependent Cytolysins
Source: Toxins (Basel). 2026 Jan 16;18(1):46. doi: 10.3390/toxins18010046 (PMC12846037; doi:10.3390/toxins18010046)
Supplement: Supplementary file 1 [file toxins-18-00046-s001.zip › toxins-4070137-supplementary.pdf]

Supplementary Materials: Functional Dissection of *Leishmania major* Membrane Components in Resistance to Cholesterol-Dependent Cytolysins

Chaitanya S. Haram, Sebastian J. Salinas, Coleman Wilson, Salma Waheed Sheikh, Kai Zhang and Peter A. Keyel

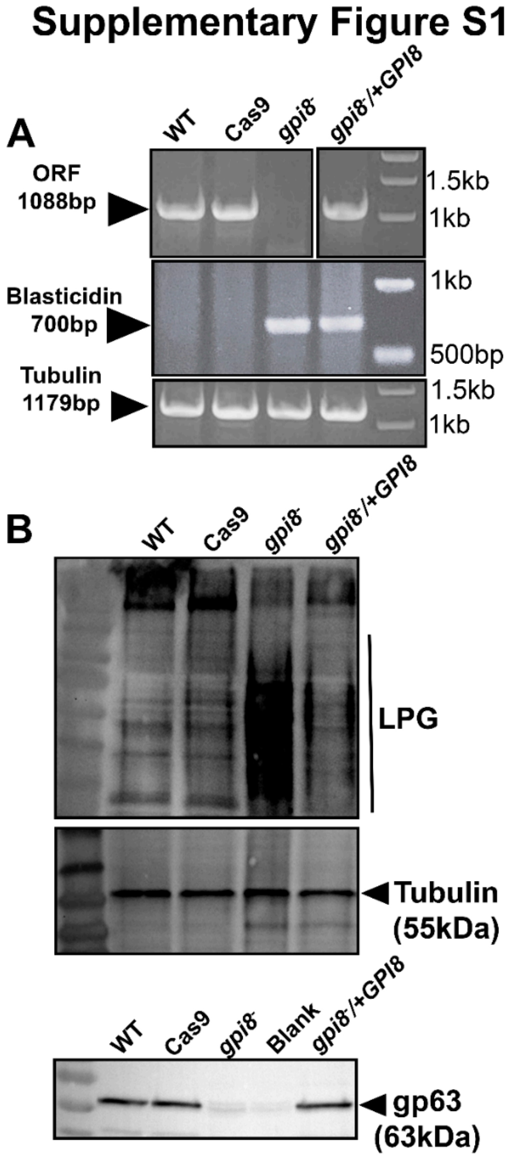

Figure S1. Generation of the *gpi8*⁻ and *gpi8*⁻/+GPI8 *L. major* strains. (A) Genomic DNA was extracted after 10-15 days of transfection from LV39WT, LV39 Cas9, *gpi8*⁻, and *gpi8*⁻/+GPI8 promastigotes and analyzed by PCR using primers for the open reading frame (1088bp), Blastidicin (700bp), or alpha tubulin (1179bp) on 1% agarose gel. (B-D) Total protein lysates were used to validate the functionality of *gpi8*. Blots were probed with the indicated antibodies. Blot is representative of 3 independent blots.

Supplementary Figure S2

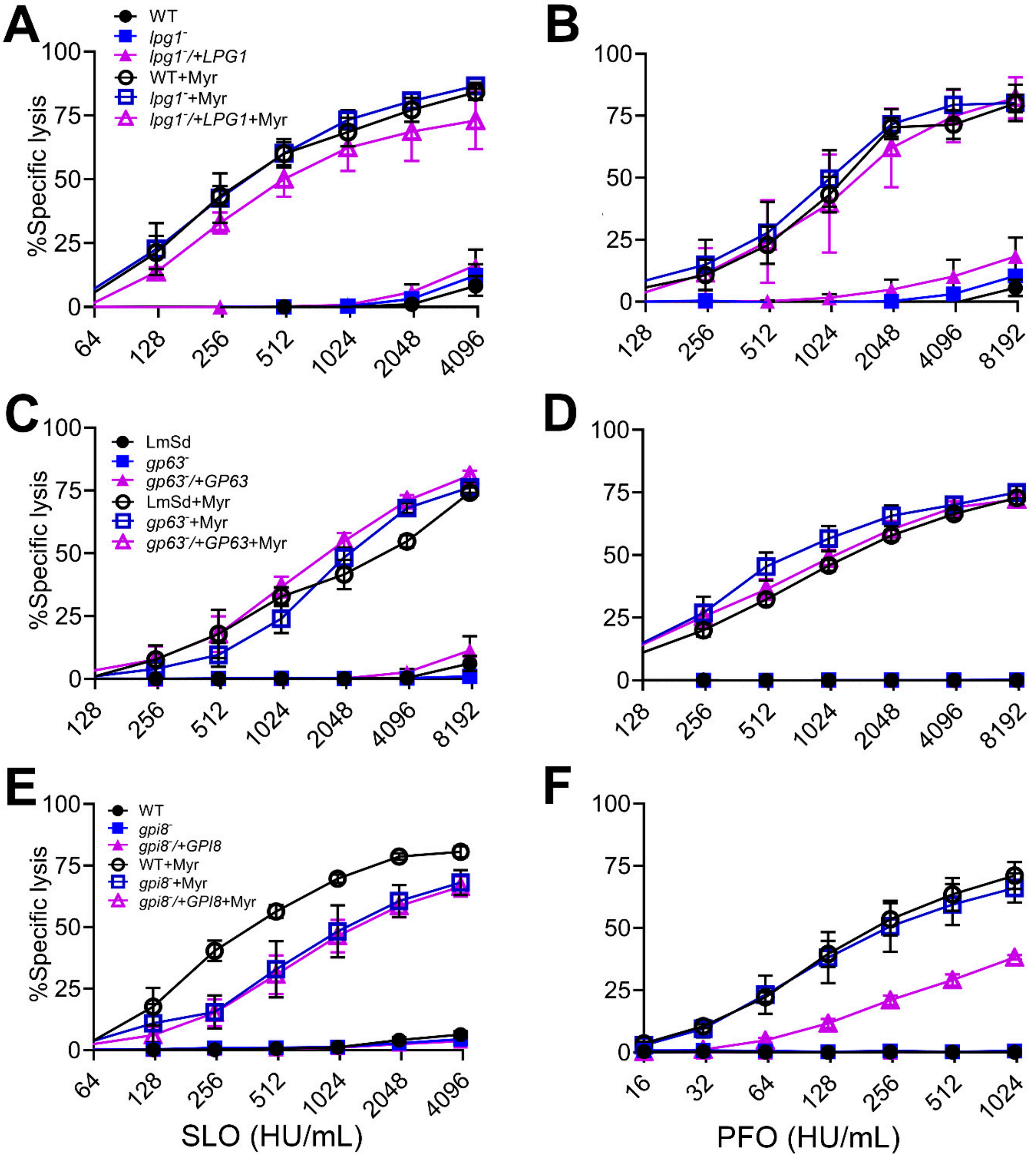

Figure S2. GPI null mutants of *L. major* are resistant to CDC cytotoxicity. LV39 wild-type (LV39WT), Seidman wild-type (WT (LmSd), *lpg1*−, *lpg1*−/+LPG1, *gp63*−, *gp63*−/+GP63, *gpi8*−, or *gpi8*−/+GPI8 *L. major* promastigotes grown in 1X M199 supplemented with either no myriocin or 10 μM myriocin (+Myr) were challenged with (A,C,E) SLO or (B,D,F) PFO at 37° C for 30 min. PI uptake was measured by flow cytometry. Graphs display mean ± SEM of 3 independent experiments.

.

Supplementary Figure S3

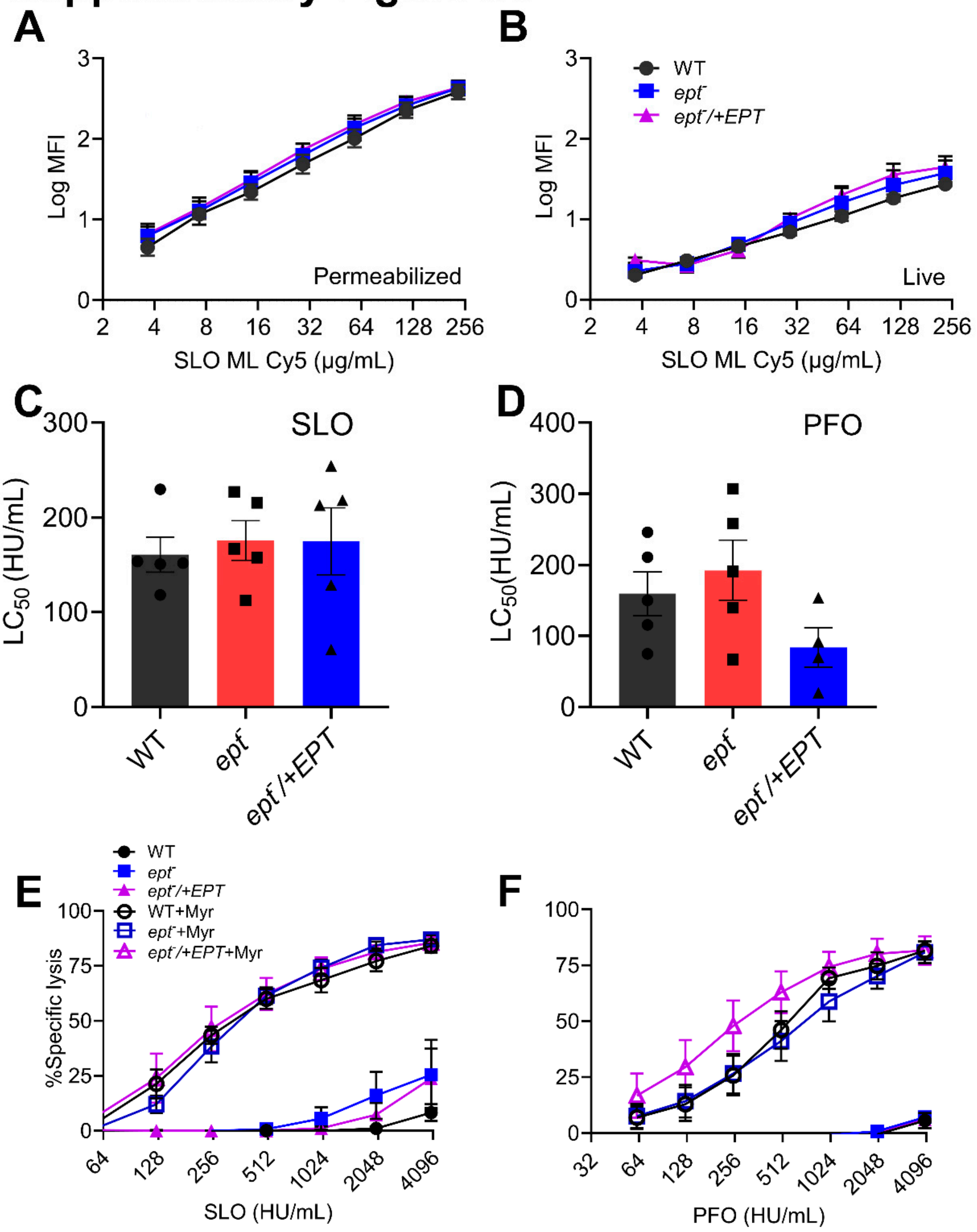

Figure S3. PME is dispensable for cytotoxicity and binding of CDCs to *L. major* promastigotes. (A, B) Wild-type (WT), *ept*−, or *ept*−/+EPT *L. major* promastigotes with or without 10 µM myriocin pretreatment were challenged with monomer-locked SLO conjugated to Cy5 at 4° C. Log median fluorescence intensity of Cy5 fluorescence gated on live and permeabilized cells is shown. (C-F) *L. major* promastigotes with or without 10 µM myriocin pretreatment were challenged with SLO (C, E) or PFO (D, F) at 37° C. PI uptake was measured by flow cytometry. (E, F) The LC_{50} values were calculated via logistic modeling. Graphs display mean ± SEM of (A, B) three or (C-F) five independent experiments, with (E, F) independent experiments plotted as individual points. Statistical significance was tested using 2-way ANOVA with multiple comparison and Sidak–Bonferroni correction.

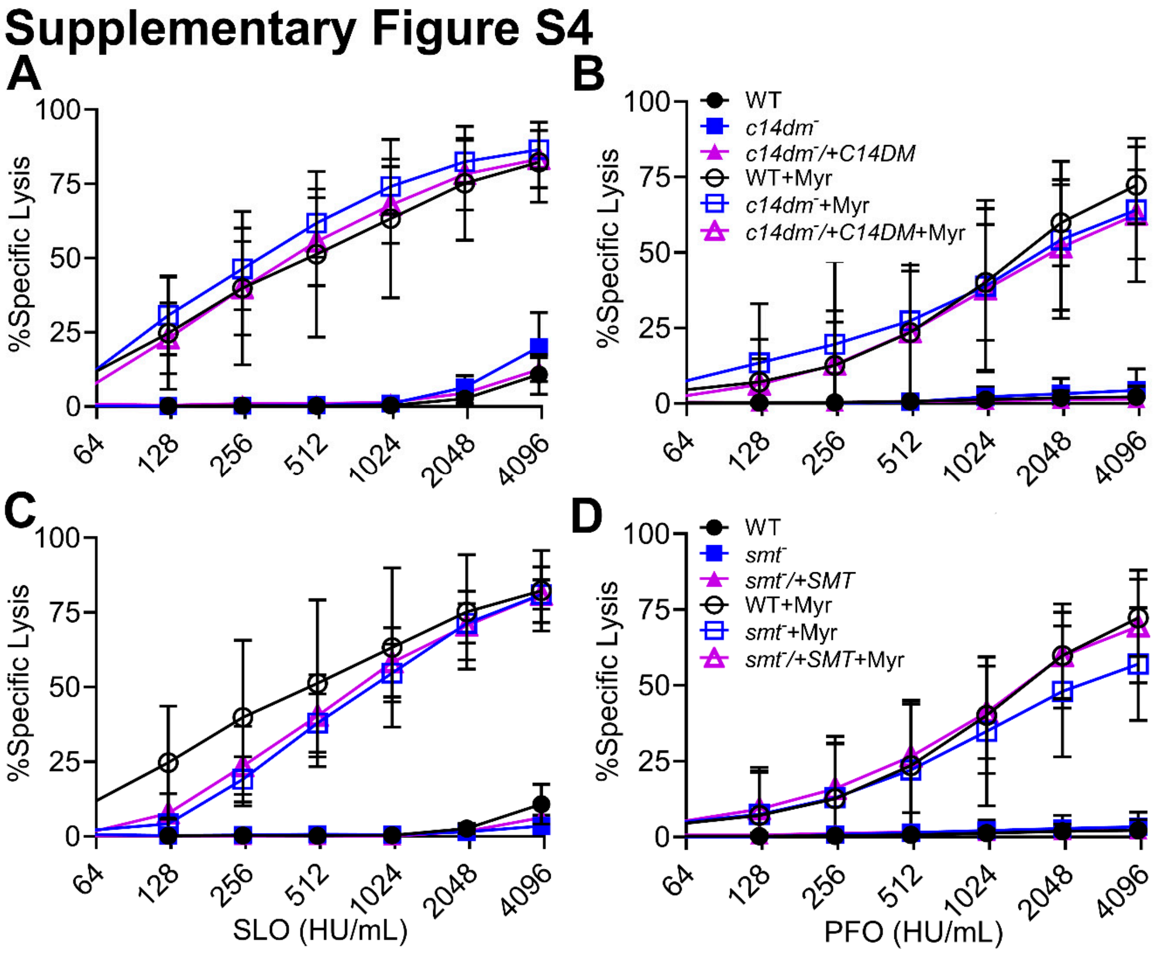

Figure S4. Ergosterol synthesis mutants *c14dm−* and *smt−* show sensitivity to CDCs only on myriocin treatment. Wild-type (WT), (A,B) *c14dm−*, *c14dm−*/+C14DM, (C, D) *smt−*, or *smt−*/+SMT promastigotes pretreated with vehicle or 10 µM myriocin were challenged with SLO or PFO at 37° C for 30 min at indicated concentrations. PI uptake was measured by flow cytometry. All genotypes were assayed together so the WT results are the same in A and C, and B and D. Graphs are split for clarity. Graphs display mean ± SEM of three independent experiments.

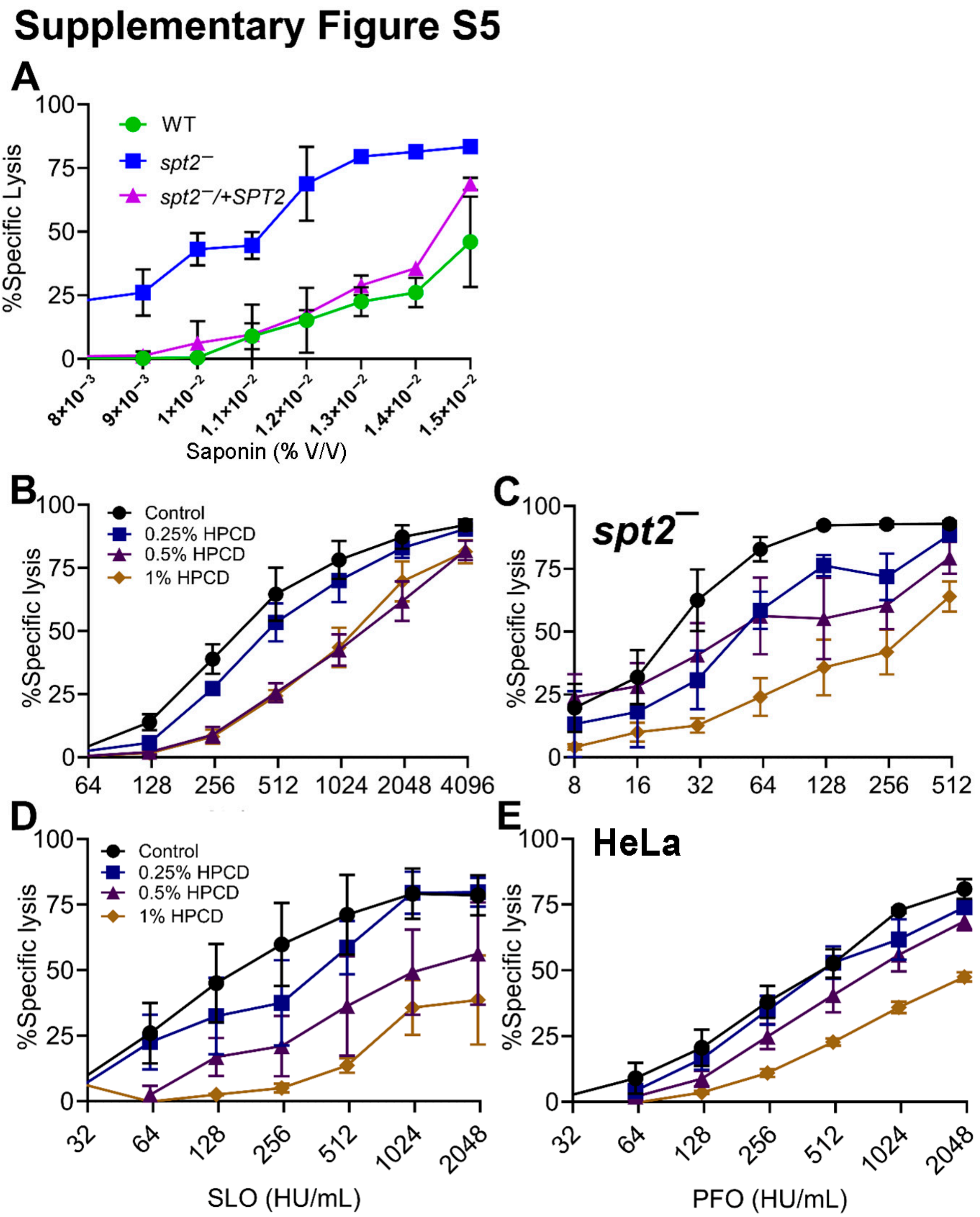

Figure S5. CDCs use sterols to lyse *L. major*. (A) Wild-type (WT), *spt2*−, and *spt2*−/+SPT2 promastigotes were challenged with the indicated concentrations of saponin (x 10^{-2} %V/V) at 37° C. PI uptake was analyzed by flow cytometry. (B, C) *L. major spt2*− or (D, E) HeLa cells pretreated with indicated concentrations of 3-hydroxylpropylcyclodextrin (HPCD) were challenged with (B,D) SLO or (C,E) PFO at 37° C for 30 min. PI uptake was measured by flow cytometry. Graphs display mean ± SEM of three independent experiments.
